# Validation of a Questionnaire Assessing the Link Between Affective State and Physical Activity in Adults: A Cross-Sectional Study

**DOI:** 10.3390/jcm14093210

**Published:** 2025-05-06

**Authors:** Constantin Ciucurel, Manuela Mihaela Ciucurel, Luminita Georgescu, Mariana Ionela Tudor, Gabriel Alexandru Olaru, Elena Ioana Iconaru

**Affiliations:** 1 Department of Medical Assistance and Physical Therapy, University Center of Pitesti, National University for Science and Technology Politehnica Bucuresti, 110040 Pitesti, Romania; constantin.ciucurel@upb.ro (C.C.); mariana.tudor0610@upb.ro (M.I.T.); 2 Department of Psychology, Communication Sciences and Social Assistance, University Center of Pitesti, National University for Science and Technology Politehnica Bucuresti, 110040 Pitesti, Romania; manuela.ciucurel@upb.ro; 3 Department of Physical Education and Sport, University Center of Pitesti, National University for Science and Technology Politehnica Bucuresti, 110040 Pitesti, Romania; lgeorgescu0507@upb.ro; 4 Doctoral School in Sport Science and Physical Education, University Center of Pitesti, National University for Science and Technology Politehnica Bucuresti, 110040 Pitesti, Romania; alexandru.tgv@yahoo.com

**Keywords:** adult population, depression, exercise motivation, physical activity level, questionnaire validation

## Abstract

**Background/Objectives:** Physical activity (PA) is a key determinant of mental and physical health, yet its relationship with affective states remains insufficiently explored. Emotional factors, such as depression, anxiety, and motivation levels, can significantly impact PA engagement. This study aims to validate the Affective State and Physical Activity Questionnaire (ASPAQ), a novel 15-item instrument designed to assess the interplay between affective states and PA in adults. **Methods:** A cross-sectional study was conducted with 412 adults (145 males, 267 females, aged 18–65 years). Participants completed the ASPAQ alongside the International Physical Activity Questionnaire—Short Form (IPAQ-SF) and the Patient Health Questionnaire-9 (PHQ-9) on an online platform, with the support of trained operators. The psychometric properties of the ASPAQ were evaluated using reliability tests (Cronbach’s alpha, McDonald’s omega), exploratory factor analysis (EFA), and correlational analyses to assess convergent validity. **Results:** The ASPAQ demonstrated excellent reliability (Cronbach’s alpha = 0.973; McDonald’s omega = 0.973) and a unidimensional structure. Convergent validity was supported by significant correlations between ASPAQ scores and established measures of PA (IPAQ-SF) and depression (PHQ-9). EFA confirmed a single-factor model, reinforcing its conceptual integrity. **Conclusions:** The ASPAQ is a reliable and valid instrument for assessing the relationship between affective states and PA. Its integration with established measures offers a comprehensive tool for evaluating emotional barriers to PA. Future studies should explore its predictive validity and potential applications in clinical and public health settings to inform personalized interventions promoting PA among individuals with affective challenges.

## 1. Introduction

Physical activity (PA) is an important promoter of both somatic and mental health, yet its relationship with emotional well-being remains complex and not fully understood. While the beneficial effects of PA on mental health, particularly in alleviating symptoms of depression and anxiety, are well-established [1,2], the underlying emotional factors that impact an individual’s capacity to engage in PA remain a topic of ongoing research and debate. Emotional states, such as mood disorders, stress, or lack of motivation, can serve as significant barriers to regular PA, making it essential to understand how affective states influence exercise behavior [3].

Significant investments from public and private institutions reflect the growing recognition of PA interventions as a fundamental strategy for improving health outcomes [4]. In this context, recent research highlights the significance of examining emotional factors that influence an individual’s capacity to engage in PA and sport, as these affective determinants can impact the efficacy of PA interventions [5]. This research direction is the starting point for developing personalized strategies that integrate individuals’ emotional states and support sustained participation in PA.

Despite this growing interest, few instruments specifically address the link between affective states and PA, and even fewer attempt to capture this relationship in an integrated manner. Existing tools focus separately on PA levels and mental health symptoms [6] but do not provide an in-depth exploration of how emotional factors directly impact participation in PA. This gap in measurement is particularly relevant as more targeted interventions are needed to help individuals with emotional struggles overcome barriers to PA [7].

Assessment tools for affective states, such as depression or anxiety, are typically distinct from those evaluating PA, creating a dichotomy in how these interrelated aspects of health are studied. Moreover, the presence of depression can limit the accuracy of clinical information, including the objectivity of determining appropriate PA regimens, as it introduces subjective biases and impairs the respondent’s ability to provide reliable self-assessments [8]. However, the integration of these domains within a single instrument is both timely and scientifically grounded. While the relationship between affective states and PA has been established through numerous studies [9,10], including in individuals with disabilities [11], current approaches often fall short in capturing the dynamic interplay between these factors. By merging these assessments, researchers can transcend the traditional paradigm of isolated evaluations, enabling a more holistic understanding of how emotional well-being directly impacts PA engagement. This innovative approach not only reflects the complexity of mind–body interactions but also aligns with the growing need for personalized, multidimensional strategies in health promotion.

This study aims to validate a newly developed questionnaire that assesses the relationship between affective states and PA in adults. Through a cross-sectional approach, this research seeks to explore how different emotional states, such as depression, anxiety, and low motivation, influence PA levels. By integrating these factors into a single instrument, this study offers a novel perspective on the complex interplay between mental and physical health. The findings could provide valuable insights for developing more effective interventions to promote PA, especially for individuals struggling with emotional challenges.

Although studies consistently highlight the positive impact of PA on mood, it remains unclear to what extent this effect is exclusive to recreational activity [12]. Moreover, less is known about the inverse relationship—how emotional states may in turn affect engagement in PA. Our questionnaire was specifically developed to address this gap by providing a nuanced understanding of how affective states influence participation in different types and contexts of PA. However, it is important to note that our instrument was not designed to directly measure the mental health benefits of PA or to explore the dose–response relationship between PA and mental health. Rather, its focus is on understanding the psychological barriers and facilitators that influence engagement in PA.

In summary, this research addresses an important gap in the current literature by creating and validating a tool that connects mental health and PA. The results could have significant implications for both clinical practices and public health strategies, offering a more comprehensive approach to promoting overall well-being. Thus, given the importance of evaluating the effectiveness of PA interventions in cases of depression [13], the instrument could provide a robust framework for quantifying integrated data on affective states and PA, enabling more precise and evidence-based assessments.

## 2. Materials and Methods

### 2.1. Study Design

This cross-sectional exploratory study aimed to validate a newly developed questionnaire designed to assess the relationship between affective states and PA in adults aged 18–65 years. The study focused on evaluating the instrument’s reliability and validity, with a particular emphasis on its ability to capture the interplay between emotional factors, such as depression and anxiety, and PA engagement. This design enables the identification of patterns and associations without inferring causation, providing a solid foundation for future longitudinal or interventional research. The reporting of this cross-sectional study follows the STROBE (Strengthening the Reporting of Observational Studies in Epidemiology) guidelines (https://www.strobe-statement.org, accessed on 23 April 2025). The completed checklist (S1) is available in the Supplementary Materials.

### 2.2. Study Population and Settings

The study was conducted on a sample of 412 adults aged 18 to 65 years (145 male and 267 female), all residing in Romania. Initially, 496 participants were recruited for the study, but 84 were excluded due to incomplete responses or outliers. Outliers were defined as responses that deviated significantly from expected patterns or showed inconsistencies. Participants were recruited using a convenience sampling method, based on their accessibility and willingness to participate, with the support of trained student operators, each of whom assisted 5–10 individuals in completing the online questionnaire. Operators provided a brief orientation to ensure participants understood the items and could respond accurately. Inclusion criteria were as follows: age between 18 and 65 years, residence in Romania, ability to provide informed consent, and the capacity to complete the questionnaire with guidance from a trained operator.

Although no formal psychiatric screening was conducted, participants with major psychiatric disorders, severe cognitive deficits, or other impairments that could hinder comprehension of the questionnaire items were excluded, based on the judgment and observations of the trained operators during the recruitment process [14]. This approach aimed to enhance data quality while maintaining inclusivity. Ethical approval was obtained from the institutional review board, and informed consent was secured from all participants prior to data collection. This online administration ensured a streamlined and standardized process, allowing for broad participation across diverse demographics.

### 2.3. Study Size

To determine the minimum required sample size, we conducted an a priori power analysis using G*Power Version 3.1.9.4 [15], based on a linear multiple regression model (fixed model, R^2^ increase), assuming 15 predictors (15 items), a medium effect size (f^2^ = 0.15), α = 0.05, and a statistical power of 0.95. This resulted in a minimum requirement of 199 participants. However, we recruited a larger sample (*n* = 496) to anticipate potential exclusions due to incomplete responses, non-compliance, or outliers. After applying exclusion criteria, the final sample comprised 412 participants. This approach ensured robust statistical power and improved stability of the psychometric estimates, particularly in exploratory factor analysis (EFA) and convergent validity testing.

### 2.4. Data Sources/Measurement

Data collection involved the administration of the Affective State and Physical Activity Questionnaire (ASPAQ), a newly developed instrument consisting of 15 items, which was originally developed in Romanian. The ASPAQ, validated in the Romanian version and with an English translation, is provided as Supplementary Material (File S2). The ASPAQ was specifically designed to assess how various affective states, such as low motivation, sadness, or anxiety, have influenced engagement in PA over the past two weeks. Each item was rated on a five-point Likert scale with clearly defined score categories: 0—Never, 1—Rarely, 2—Sometimes, 3—Often, 4—Always.

The ASPAQ was developed using a structured approach, starting with a comprehensive literature review to identify key dimensions linking affective states (e.g., depression, anxiety, low motivation) with PA. Focus groups with experts in psychology and physiotherapy provided theoretical and practical insights, guiding the creation of 21 preliminary items. These were iteratively reviewed for clarity, relevance, and alignment with the identified dimensions. This structured development process was designed to ensure the content validity of the ASPAQ, ensuring that it comprehensively and accurately captures the theoretical constructs it aims to measure. Expert feedback ensured that the final set of 15 items was both accessible and meaningful, effectively capturing the interplay between emotional states and PA engagement. For this questionnaire, the total score ranges from 0 to 60, and four depression classes were proposed (none, mild, moderate, and severe) in relation to PA involvement.

To ensure robust validation of the ASPAQ, two standardized instruments were administered consecutively alongside it: the International Physical Activity Questionnaire—Short Form (IPAQ-SF), which assesses PA levels, and the Patient Health Questionnaire-9 (PHQ-9), which evaluates depressive symptoms. The IPAQ-SF is a well-established tool recognized for its reliability and validity in quantifying PA across diverse populations. It provides a structured approach to measuring the frequency, duration, and intensity of activities across multiple domains, making it an essential resource for both clinical applications and research studies [16,17,18]. The IPAQ is licensed under CC BY 4.0, details: https://creativecommons.org/licenses/by/4.0/ (accessed on 28 February 2025). The IPAQ-SF assesses PA over the past seven days in terms of frequency, duration, and intensity across domains such as work, transport, household activities, and leisure. Finally, it calculates MET (Metabolic Equivalent of Task) in minutes per week and estimated calorie expenditure, categorizing activity levels as low, moderate, or high [19].

The Patient Health Questionnaire-9 (PHQ-9) is a widely utilized, publicly available instrument designed to assess depressive symptoms based on the DSM-5 criteria for Major Depressive Disorder (MDD) [20]. This self-administered tool evaluates the prevalence and severity of nine core depressive symptoms over the preceding two weeks using a four-point response scale: 0 (not at all), 1 (several days), 2 (more than half the days), and 3 (nearly every day) [21].

The PHQ-9 is highly reliable and demonstrates robust psychometric properties for use in clinical and research settings. A cut-off score of ≥10 has been shown to detect MDD [22,23]. Scores are categorized as follows: 0–4 (absent/minimal depression), 5–9 (mild depression), 10–14 (moderate depression), 15–19 (moderately severe depression), and 20–27 (severe depression) [22]. This makes the PHQ-9 a valuable tool for both screening and monitoring depressive symptoms in diverse populations.

The three questionnaires were administered online in a fixed sequence—IPAQ-SF, PHQ-9, and ASPAQ—using the PsyToolkit platform https://www.psytoolkit.org/ (accessed on 28 February 2025), which enabled efficient and standardized online data collection [24,25]. All instruments were administered in Romanian, using linguistically adapted versions to ensure clarity and cultural appropriateness for the target population. By correlating the ASPAQ scores with those of the IPAQ-SF and PHQ-9, the study aimed to establish convergent validity, evaluating whether the ASPAQ aligns with theoretical constructs (e.g., affective states and PA) measured by well-established instruments. Participants completed the questionnaires under the supervision of trained operators who provided standardized instructions to ensure clarity and accuracy.

### 2.5. Variables and Statistical Methods

The primary variables assessed in this study included age, sex, height (H), weight (W), MET and IPAQ-SF category, total score and depression level category through the PHQ-9 scale, as well as ASPAQ score and ASPAQ category. H and W were self-reported, a method shown to be valid for collecting anthropometric data in online surveys [26]. Descriptive statistics were computed for all variables, including frequency distributions, means, and standard deviations (SDs), to provide an overview of the sample characteristics.

The reliability of the ASPAQ was assessed using both Cronbach’s alpha and McDonald’s omega. While Cronbach’s alpha is commonly used to measure internal consistency, McDonald’s omega was also calculated to provide a more robust estimate of reliability, particularly for multidimensional constructs. Omega offers a more accurate assessment of reliability by better accounting for item-level variance, making it a complementary measure to Cronbach’s alpha [27].

To assess convergent validity, both parametric and non-parametric correlation methods were utilized, with the choice depending on the distribution of the data. Parametric correlations were calculated using Pearson’s correlation coefficient, while Kendall’s tau correlation coefficient was used when the data did not meet the assumptions of normality. Normality was tested using the Shapiro–Wilk test to determine the appropriate correlation method. Statistical significance was set at a two-tailed *p*-value of <0.05 for all tests. These methods were used to establish the relationship between the ASPAQ and established measures of PA (IPAQ-SF) and depression (PHQ-9), providing evidence of the construct validity of the ASPAQ.

The analysis was completed using EFA to identify the underlying structure of the data and determine whether the questionnaire measures a single construct or multiple dimensions. The methods were selected based on the distribution of the data, as assessed by the Shapiro–Wilk test, to ensure the appropriate approach was used for the analysis. Statistical analysis of the data was performed using IBM SPSS 26.0 software (IBM Corp., Armonk, NY, USA) [28].

## 3. Results

The analysis focused on the ASPAQ, evaluating its reliability, validity, and ability to capture the relationship between affective states and PA engagement. Overall, the ASPAQ items were designed to examine the complex relationship between affective states and PA, focusing on key domains such as motivation, emotional barriers, and the influence of psychological well-being on exercise engagement. The questionnaire aims to identify how symptoms of depression, anxiety, and low mood—such as lack of energy, reduced interest, and negative self-perception—affect participation in and enjoyment of PA. Additionally, it explores the role of emotional factors like stress, self-criticism, and challenges in group settings in promoting sedentary behaviors or impeding an active lifestyle. By capturing these dynamics, the ASPAQ offers a comprehensive assessment of how affective states shape PA engagement, providing insight into emotional barriers to exercise.

For this questionnaire, the total score ranges from 0 to 60, with four ASPAQ categories established to reflect the impact of affective states PA: minimal, mild, moderate, and severe impact. These categories were defined using percentiles, with the 25th, 50th, and 75th percentiles as cutoffs. Scores below the 25th percentile represent minimal impact, scores between the 25th and 50th percentiles indicate mild impact, scores between the 50th and 75th percentiles reflect moderate impact, and scores above the 75th percentile correspond to severe impact. This data-driven approach classifies the sample based on score distribution, resulting in the following intervals: 0 to 10 (minimal impact), 11 to 31 (mild impact), 32 to 37 (moderate impact), and above 38 (severe impact). It should be noted that these categories are exploratory in nature and should not be interpreted as diagnostic criteria.

Descriptive statistics for demographic data, scores from the three questionnaires, and participant distribution across the categories of each questionnaire are presented in Table 1 and Table 2.

The reliability analysis of the ASPAQ showed high internal consistency, with Cronbach’s alpha at 0.973 and McDonald’s omega at 0.973. These results confirm the reliability of the instrument in accurately measuring the relationship between affective states and PA. The ‘if item deleted’ analysis showed no redundant items, as removing any item did not increase Cronbach’s alpha, confirming the contribution of each item to the scale’s consistency.

To examine the convergent validity of the ASPAQ, we conducted a correlational analysis to explore the relationships between variables extracted from IPAQ-SF, PHQ-9, and ASPAQ. Since the Shapiro–Wilk test indicated a non-normal distribution of the data, we utilized Kendall’s tau correlation coefficient, a non-parametric measure suitable for such conditions. Statistical significance was also calculated to assess the relevance of the observed correlations. To facilitate the identification of patterns and relationships, a correlation matrix was created to display the associations between key variable pairs (see Table 3).

According to correlation classification [29], the results indicated moderate to strong, statistically significant correlations. The results show moderate to strong, statistically significant correlations (*p* < 0.01), which is expected given the conceptual differences between these instruments, between the variables derived from the ASPAQ and those from the IPAQ-SF and PHQ-9. These findings suggest that the ASPAQ effectively captures aspects of affective states and PA that overlap with constructs measured by established tools. This alignment supports its convergent validity, implying that the ASPAQ can be confidently used as a complementary or alternative instrument for assessing the interplay between emotional states and PA, particularly in contexts where these dimensions are critical for understanding health behaviors and psychological well-being.

The final analysis focused on EFA, which aimed to identify the underlying structure of the data. Since the item scores were not normally distributed, Principal Axis Factoring (PAF) was chosen as the extraction method. This approach is particularly suitable for non-normally distributed data, enabling the identification of underlying latent variables.

First, we ensured that the sample was adequate for factor analysis. The Kaiser–Meyer–Olkin (KMO) measure of sampling adequacy was 0.973, indicating excellent factorability. Additionally, Bartlett’s Test of Sphericity was significant, χ^2^(105) = 6540.24, *p* < 0.001, confirming that the correlation matrix was appropriate and that there were enough meaningful correlations between the items to justify applying the EFA.

**Figure 1:**
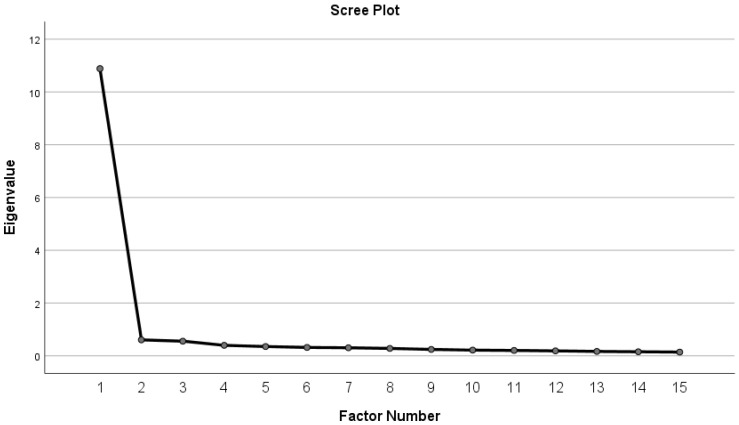
Scree plot for factor extraction.

Next, we proceeded with the extraction of the factors using Principal Axis Factoring (PAF). The factor rotation method chosen was Oblimin rotation, assuming that the items might be correlated. This is a common assumption in many psychological and social science questionnaires, where the items often share a relationship. However, this assumption can be revisited if needed, based on the results. To determine the optimal number of factors to extract, we referred to the scree plot and considered Eigenvalues greater than 1. The obtained scree plot (Figure 1) clearly showed a sharp ‘elbow’ after the first factor, which is a common indicator of the unidimensionality of the data, confirming that a single factor adequately represents the latent structure of the questionnaire.

Finally, factor scores for each participant were saved as new variables. These scores represent the extent to which each participant exhibits the underlying latent factor identified by the analysis and can be used in further analyses or for validation purposes. As shown in Table 4, all 15 items exhibit factor loadings above 0.6, demonstrating strong contributions to the extracted factor and reinforcing the unidimensional structure of the questionnaire.

In summary, the EFA revealed that the ASPAQ questionnaire is unidimensional, with all 15 items loading strongly onto a single factor, further confirming the robustness of the single-factor solution in measuring a unified construct.

## 4. Discussion

The primary objectives of this study were to evaluate the reliability and convergent validity of the ASPAQ questionnaire, aiming to establish its effectiveness in assessing the relationship between affective states and PA. Specifically, we sought to confirm its internal consistency, examine its convergent validity by correlating it with established measures such as the IPAQ-SF and PHQ-9, and explore its underlying structure using EFA. These steps were integral to demonstrating the questionnaire’s robustness as a reliable and valid instrument for research and practical applications.

A comprehensive interpretation of the results requires an initial examination of the participants’ demographic and clinical characteristics. The study sample comprised adults aged 18 to 65 years, with a mean age of 37.81 ± 13.05 years. Based on a PHQ-9 cutoff score of 10, 7.7% of participants were classified as being at high risk for MDD. Regarding PA levels, as assessed using the IPAQ-SF, the majority of participants (56.1%) exhibited low PA, 27.2% engaged in moderate PA, and 16.7% reported high PA levels.

The prevalence of MDD within our study population aligns with prior epidemiological estimates. For instance, a previous study identified a 6.7% prevalence of depression in the general adult population aged 19 years and older, utilizing a PHQ-9 cutoff of ≥10 [30]. However, there remains an ongoing discourse in the scientific literature concerning the optimal PHQ-9 threshold for detecting depression. Some researchers contend that a threshold of ≥10 may lead to an overestimation of depression prevalence, advocating instead for a more conservative cutoff, such as ≥14, to enhance diagnostic specificity. Despite these variations in cutoff recommendations, it is essential to recognize that the PHQ-9 serves as a screening instrument rather than a diagnostic tool. Consequently, its results should not be employed as a substitute for structured clinical interviews in establishing the prevalence of major depression [31]. Furthermore, meta-analyses indicate that when semi-structured interviews are utilized, the estimated prevalence of major depression is approximately 14% [32].

The next aspect under discussion is the high reliability of ASPAQ. Demonstrated through excellent values of Cronbach’s alpha and McDonald’s omega, (0.973), this reliability highlights the instrument’s robustness and precision. Such results indicate that ASPAQ consistently measures the relationship between affective states and PA, ensuring its suitability for both research and clinical applications. The strong internal consistency of the tool provides confidence in its ability to deliver reliable insights, a critical factor for advancing evidence-based practices. Moreover, ASPAQ fills a notable void in existing assessment tools, which often measure affective states and PA independently. This integrative approach offers a more comprehensive understanding of the dynamic interplay between emotional well-being and PA. By bridging this methodological gap, ASPAQ significantly contributes to the field, opening new avenues for research and targeted interventions aimed at improving both mental and physical health outcomes.

Previous research efforts have primarily concentrated on developing instruments to assess how PA influences affective states, aiming to capture the emotional responses induced by exercise [33]. However, our study takes a different approach by focusing on the impact of affective states on PA engagement, addressing a gap in the existing literature. This shift in focus provides a complementary perspective, recognizing the role of emotional states as predictors of PA participation, rather than just as outcomes.

Another important point is that other researchers have primarily focused on assessing the acute effects of PA on affective states, using multidimensional instruments designed to capture the emotional responses induced by exercise [34], sometimes in depressive subjects compared to healthy control groups [35]. In contrast, our instrument stands out due to its unidimensional approach, as confirmed by the EFA, which specifically examines the impact of affective states on engagement in PA, and not the immediate effects of exercise on affect. This conceptual difference provides a clearer understanding of how affective states influence PA behaviors.

Building on this conceptual difference, our findings resonate with recent research that explores the influence of both affective and instrumental outcomes on motivation and PA engagement, which demonstrates the role of positive affect and weight loss beliefs in enhancing PA behavior [36]. However, our approach offers a distinct perspective by focusing specifically on how affective states shape engagement in PA, independent of instrumental motivations. This differentiation also highlights the significance of emotional states as a primary factor in motivating sustained PA, suggesting that interventions targeting emotional well-being could enhance long-term PA participation.

While previous critiques have questioned the conceptual clarity and practical utility of instruments measuring exercise-induced affective responses—particularly regarding the ambiguous definition of “feeling” and the redundancy of certain subscales [37]—these concerns do not apply to ASPAQ. Unlike such measures, which assess how PA influences affective states, ASPAQ takes the reverse approach by evaluating how affective states impact engagement in PA. This fundamental difference ensures that the conceptual ambiguities and methodological concerns raised in prior critiques are not relevant to our instrument, as its primary focus lies in understanding the motivational role of affect rather than capturing post-exercise emotional states.

In summary, while existing questionnaires primarily focus on how PA interventions influence affective states by capturing exercise-induced emotional changes and demonstrate sensitivity to experimental variations in PA [38], ASPAQ offers an innovative perspective by assessing how affective states shape motivation and intention to engage in PA.

Another aspect that needs discussion is the correlation between ASPAQ scores and categories with PHQ-9 and IPAQ-SF. In general, construct validity is supported by significant correlations between an instrument and other already validated constructs that are conceptually similar [39]. Our results show moderate to strong, statistically significant correlations (*p* < 0.01), which is expected given the similarities, but also the conceptual differences, between these instruments. ASPAQ specifically measures the relationship between affectivity and PA, whereas PHQ-9 and IPAQ-SF assess depressive symptoms and activity levels more broadly. These correlations confirm the convergent validity of ASPAQ, indicating that it effectively captures the influence of affective states on PA. The moderate to strong correlations reassure us that ASPAQ provides complementary insights without fully overlapping with the constructs measured by PHQ-9 and IPAQ-SF, reinforcing its relevance as a specific tool for studying the affectivity–PA relationship.

Although the study relied on a convenience sample of Romanian adults, the relatively large sample size and the demographic variability in age, gender, and physical activity level enhance the external validity of the findings within similar populations. The online mode of administration further enabled wide geographic reach, supporting broader applicability within the Romanian context. However, generalizing these findings to other countries or cultural settings should be carried out with caution, as factors such as language, health behaviors, and socio-cultural norms may influence the relationship between affective states and PA. Future research should explore cross-cultural adaptations of the ASPAQ to confirm its utility in diverse populations.

Limitations: Despite the valuable insights provided by this study, several limitations should be acknowledged. First, the cross-sectional design limits the ability to establish causal relationships between affective states and PA. Additionally, since the questionnaires were administered online, self-reported responses may be affected by biases, such as social desirability, and by technical issues, like connection interruptions, which could impact accuracy [40,41]. The study also relied on convenience sampling, which may affect the generalizability of the findings to broader populations. Lastly, while the Shapiro–Wilk test revealed non-normality in the data, further testing with other distributions could provide a more nuanced understanding of the data’s characteristics. In addition, the absence of a confirmatory factor analysis (CFA) represents a methodological limitation, as it prevents more rigorous validation of the factor structure identified through EFA. Future research should address this by applying CFA to independent samples. Moreover, anxiety symptoms were not specifically assessed, which may represent a potential confounding factor given their frequent co-occurrence with depressive symptoms.

Strengths of the study: Our research presents a novel contribution to the field through the development and psychometric evaluation of the ASPAQ, a new questionnaire designed to assess the relationship between affective states and PA. The rigorous application of statistical techniques, including Cronbach’s alpha, McDonald’s omega, and EFA, ensures robust reliability and validity. The findings highlight the unidimensional structure of the ASPAQ, reinforcing its capacity to capture key emotional states related to PA. By using standardized instruments like the IPAQ-SF and PHQ-9, this study demonstrates the ASPAQ’s convergent validity and its potential to provide valuable insights into the connection between affective states and PA.

Beyond its research relevance, the ASPAQ holds promise for clinical applications by offering a rapid, self-administered tool to evaluate how affective states influence PA behavior. In healthcare settings, it could serve as a screening instrument to identify individuals at risk of physical inactivity due to emotional distress or low motivation, complementing existing measures such as PHQ-9 and IPAQ-SF. Its integration into routine assessments may support the development of personalized interventions that address both psychological and behavioral dimensions, particularly in primary care, physiotherapy, mental health, or lifestyle medicine contexts.

## 5. Conclusions

The present study validated the ASPAQ as a reliable and conceptually coherent instrument for assessing how affective states influence PA engagement. The ASPAQ captures emotional and psychological barriers that may reduce participation in PA. This tool may be particularly useful in clinical settings, where rapid and accessible screening instruments are needed to identify individuals at risk of physical inactivity due to affective distress. It supports a more comprehensive understanding of the interplay between emotional well-being and PA patterns and may inform the development of personalized interventions that integrate both psychological and behavioral dimensions. Although the instrument demonstrated strong psychometric properties, further validation through CFA and test–retest reliability is recommended to ensure its applicability in broader and more diverse populations.

## Figures and Tables

**Table 1:** Descriptive summary of participant characteristics, including parametric variables (*n* = 412).

Variable	Age (Years)	W (kg)	H (cm)	IPAQ-SF MET	PHQ-9 Score	ASPAQ Score
mean	37.81	67.15	167.66	1451.95	6.90	25.89
SD	13.05	12.16	7.63	1686.12	3.72	14.80

Note—W: weight; H: height; IPAQ-SF: International Physical Activity Questionnaire—Short Form; PHQ-9: Patient Health Questionnaire-9; ASPAQ: Affective State and Physical Activity Questionnaire; MET: Metabolic Equivalent of Task in minutes per week; SD: standard deviation; *n*: number of subjects.

**Table 2:** Distribution of participant characteristics and group classifications (*n* = 412).

Variable	Category	Frequency (*n*)	Percentage (%)
Sex	Male	145	35.2
	Female	267	64.8
IPAQ-SF Category	Low PA	231	56.1
	Moderate PA	112	27.2
	High PA	69	16.7
PHQ-9 Category	Absent/Minimal depression (0–4)	112	27.2
	Mild depression (5–9)	268	65
	Moderate depression (10–14)	22	5.3
	Moderately severe depression (15–19)	7	1.7
	Severe depression (20–27)	3	0.7
ASPAQ Category	Minimal impact (0–15)	99	24
	Mild impact (16–30)	103	25
	Moderate impact (31–45)	104	25.2
	Severe impact (45–60)	106	25.7

Note—IPAQ-SF: International Physical Activity Questionnaire—Short Form; PHQ-9: Patient Health Questionnaire-9; ASPAQ: Affective State and Physical Activity Questionnaire; PA: physical activity; *n*: number of subjects.

**Table 3:** Matrix of correlations (Kendall’s tau correlation coefficient, τ) between recorded variables and level of statistical significance, *p* (*n* = 412).

Variable	MET	IPAQ-SF Category	PHQ-9 Score	PHQ-9 Category	ASPAQ Score	ASPAQ Category
MET	1.00					
IPAQ-SF category	0.736 *	1.00				
PHQ-9 score	−0.385 *	−0.565 *	1.00			
PHQ-9 category	−0.327 *	−0.517 *	0.766 *	1.00		
ASPAQ score	−0.397 *	−0.595 *	0.617 *	0.579 *	1.00	
ASPAQ category	−0.444 *	−0.658	0.650 *	0.628 *	0.878 *	1.00

Note—IPAQ-SF: International Physical Activity Questionnaire—Short Form; PHQ-9: Patient Health Questionnaire-9; ASPAQ: Affective State and Physical Activity Questionnaire; MET: Metabolic Equivalent of Task in minutes per week; *: *p* < 0.01 was considered statistically significant (2-tailed); *n*: group size.

**Table 4:** Factor matrix for the ASPAQ (Principal Axis Factoring, 1 factor extracted, 3 iterations required).

Item 1	Item 2	Item 3	Item 4	Item 5	Item 6	Item 7	Item 8	Item 9	Item 10	Item 11	Item 12	Item 13	Item 14	Item 15
0.822	0.900	0.855	0.844	0.851	0.829	0.864	0.877	0.852	0.882	0.843	0.862	0.676	0.801	0.827

## Data Availability

The data are available on request from the corresponding author. All data relevant to the study are included in the article.

## Supplementary Materials

The following supporting information can be downloaded at https://www.mdpi.com/article/10.3390/jcm14093210/s1: Checklist S1: STROBE checklist for cross-sectional studies; File S2: Full version of the ASPAQ questionnaire in Romanian and English.
