# Supplementary material for: Validation of a Questionnaire Assessing the Link Between Affective State and Physical Activity in Adults: A Cross-Sectional Study
Source: J Clin Med. 2025 May 6;14(9):3210. doi: 10.3390/jcm14093210 (PMC12072974; doi:10.3390/jcm14093210)
Supplement: Supplementary file 1 [file jcm-14-03210-s001.zip › Checklist S1.pdf]

STROBE Statement—Checklist of items that should be included in reports of ***cross-sectional studies***

	Item No	Recommendation	Comments and quotes	Page
**Title and abstract**	1	(*a*) Indicate the study's design with a commonly used term in the title or the abstract	The title explicitly states the study design as "a Cross-Sectional Study", which adheres to reporting guidelines.	1
		(*b*) Provide in the abstract an informative and balanced summary of what was done and what was found	The abstract provides a structured and balanced summary, including background, objectives, methods, results, and conclusions.	1
**Introduction**				
Background/rationale	2	Explain the scientific background and rationale for the investigation being reported	The introduction explains the lack of integrated instruments that assess the relationship between affective states and physical activity. The rationale is based on the need for a tool that captures how emotional variables influence engagement in physical activity, as distinct from tools that measure either affect or physical activity separately.	1-3
Objectives	3	State specific objectives, including any prespecified hypotheses	The manuscript clearly states the primary objective: to validate the Affective State and Physical Activity Questionnaire (ASPAQ), a novel tool designed to assess the relationship between affective states and physical activity engagement in adults. No prespecified hypotheses are explicitly mentioned	2
**Methods**				
Study design	4	Present key elements of study design early in the paper	A cross-sectional exploratory design is described in the Materials and Methods section, used to validate a new questionnaire assessing the link between affective states and physical activity. The design supports identification of associations without implying causality.	3
Setting	5	Describe the setting, locations, and relevant dates, including periods of recruitment, exposure, follow-up, and data collection	Data were collected online across Romania in early 2025 using a standardized online platform. Participants were guided by trained operators during a single assessment session; no follow-up was conducted due to the cross-sectional design.	3
Participants	6	(*a*) Give the eligibility criteria, and the sources and methods of selection of participants	Adults aged 18–65 residing in Romania were eligible. Participants were recruited through convenience sampling, with data collection supported by trained operators who assisted individuals in completing the online questionnaire.	3

Variables	7	Clearly define all outcomes, exposures, predictors, potential confounders, and effect modifiers. Give diagnostic criteria, if applicable	Variables include scores from the ASPAQ, PHQ-9, and IPAQ-SF, along with age, sex, height, and weight. All data were self-reported. No diagnostic criteria were applied, as the instruments are standardized questionnaires rather than clinical assessments.	4-5
Data sources/ measurement	8*	For each variable of interest, give sources of data and details of methods of assessment (measurement). Describe comparability of assessment methods if there is more than one group	Data were collected using validated instruments: ASPAQ (affective states and physical activity), IPAQ-SF (physical activity levels), and PHQ-9 (depressive symptoms). All were administered online with standardized procedures.	3-4
Bias	9	Describe any efforts to address potential sources of bias	Bias was minimized by training operators, excluding outliers, and using standardized online tools for data collection.	3-5
Study size	10	Explain how the study size was arrived at	The sample size was determined through an a priori power analysis using G*Power, assuming 15 predictors, medium effect size ($f^2 = 0.15$), $\alpha = 0.05$, and 0.95 power, which indicated a minimum of 199 participants. A larger sample (n = 496) was recruited to account for exclusions, resulting in 412 participants after applying exclusion criteria.	3
Quantitative variables	11	Explain how quantitative variables were handled in the analyses. If applicable, describe which groupings were chosen and why	Quantitative variables were analysed using descriptive statistics (means, SD), internal consistency analysis (Cronbach's alpha, McDonald's omega), and correlation methods (Kendall's tau). Exploratory Factor Analysis (EFA) was used to identify the underlying structure of the data.	4-5
Statistical methods	12	(*a*) Describe all statistical methods, including those used to control for confounding	Descriptive statistics, reliability indices (Cronbach's alpha, McDonald's omega), Kendall's tau correlation coefficient, and Exploratory Factor Analysis (EFA) were employed. No formal statistical methods were used to control for confounding, as no significant confounders were identified or specifically tracked in this study. Normality was tested using the Shapiro-Wilk test, and correlation methods were chosen based on the distribution of the data.	4-5
		(*b*) Describe any methods used to examine subgroups and interactions	Subgroup analyses were performed descriptively by classifying participants into various categories based on the scores obtained on the ASPAQ, PHQ-9, and IPAQ-SF. The primary goal of the study was to validate the ASPAQ questionnaire, and the subgroup analysis was limited to a descriptive examination of how participants were categorized into different classes,	3-5

			such as such as physical activity levels (IPAQ-SF), depression severity (PHQ-9), and ASPAQ impact levels, as well as by sex.	
		(*c*) Explain how missing data were addressed	Missing data were addressed by excluding participants with incomplete responses (n=84) prior to analysis. Outliers, defined as responses that deviated significantly from expected patterns or showed inconsistencies, were also excluded.	3
		(*d*) If applicable, describe analytical methods taking account of sampling strategy	Not applicable – no complex sampling strategy used	-
		(*e*) Describe any sensitivity analyses	No sensitivity analyses were conducted.	-
**Results**				
Participants	13*	(a) Report numbers of individuals at each stage of study—eg numbers potentially eligible, examined for eligibility, confirmed eligible, included in the study, completing follow-up, and analysed	A total of 496 participants were initially recruited. After exclusions due to incomplete responses or outliers, 412 participants were included in the analysis.	3
		(b) Give reasons for non-participation at each stage	Participants were excluded due to incomplete responses or detection of outliers, which were defined as responses deviating significantly from expected patterns or showing inconsistencies.	3
		(c) Consider use of a flow diagram	A flow diagram was not used; however, participant flow is described in the text.	3
Descriptive data	14*	(a) Give characteristics of study participants (eg demographic, clinical, social) and information on exposures and potential confounders	Descriptive data, including demographic characteristics, clinical measures, and participant classification (by sex, physical activity levels, depression severity, and affective state impact on physical activity), are presented in Table 1 and Table 2.	6
		(b) Indicate number of participants with missing data for each variable of interest	There were no missing data for any of the variables of interest. All participants had complete data, and no imputation or exclusion due to missing values was necessary.	3
Outcome data	15*	Report numbers of outcome events or summary measures	The ASPAQ, PHQ-9, and IPAQ-SF scores were summarized, showing mean scores and standard deviations, as well as the distribution of participant classifications (e.g., affective state impact on physical activity, depression severity, and physical activity levels). Correlation analyses between these measures were also performed to explore their relationships.	5-7

Main results	16	(*a*) Give unadjusted estimates and, if applicable, confounder-adjusted estimates and their precision (eg, 95% confidence interval). Make clear which confounders were adjusted for and why they were included	No confounder-adjusted models were used, as the analysis focused on exploring the bivariate associations between the measures, and no adjustments for confounders were deemed necessary	6-7
		(*b*) Report category boundaries when continuous variables were categorized	The category boundaries for PHQ-9, IPAQ-SF, and ASPAQ were clearly defined based on predefined scoring thresholds for each respective measure	4-5
		(*c*) If relevant, consider translating estimates of relative risk into absolute risk for a meaningful time period	Not applicable – not a risk-based analysis.	-
Other analyses	17	Report other analyses done—eg analyses of subgroups and interactions, and sensitivity analyses	Internal consistency analysis was performed first, followed by Exploratory Factor Analysis (EFA) to assess the reliability and underlying structure of the ASPAQ.	6-8
**Discussion**				
Key results	18	Summarise key results with reference to study objectives	The main findings of the study, which are further discussed in the Discussion and Conclusion sections, highlight the high internal consistency of the ASPAQ, with Cronbach's alpha and McDonald's omega both at 0.973, confirming its reliability. The correlation analysis revealed significant relationships between ASPAQ scores and measures of physical activity (IPAQ-SF) and depression severity (PHQ-9), supporting its convergent validity. Additionally, the Exploratory Factor Analysis (EFA) identified a unidimensional structure for the ASPAQ, further validating its use as a tool for assessing the relationship between affective states and physical activity engagement. These findings were aligned with the study's objective of validating the ASPAQ and examining its ability to capture the complex interplay between affective states and physical activity	8-11
Limitations	19	Discuss limitations of the study, taking into account sources of potential bias or imprecision. Discuss both direction and magnitude of any potential bias	This study is subject to several limitations, including the cross-sectional design, which limits causal inference, and reliance on self-reported, online data, which may be influenced by social desirability or technical issues. The use of convenience sampling may reduce generalizability, and the lack of confirmatory factor analysis (CFA) limits structural validation. Additionally, potential confounding from unmeasured anxiety symptoms cannot be ruled out.	10

Interpretation	20	Give a cautious overall interpretation of results considering objectives, limitations, multiplicity of analyses, results from similar studies, and other relevant evidence	The results were interpreted in light of the study objectives and limitations, with consideration of the multiplicity of analyses and alignment with findings from similar studies. Relevant literature was referenced to contextualize the results and support conclusions.	8-10
Generalisability	21	Discuss the generalisability (external validity) of the study results	The ASPAQ demonstrated strong psychometric properties in a Romanian adult sample, suggesting good applicability within similar populations. However, its use in other cultural or linguistic contexts requires further validation to ensure conceptual and functional equivalence.	10
**Other information**				
Funding	22	Give the source of funding and the role of the funders for the present study and, if applicable, for the original study on which the present article is based	Funded by the National University of Science and Technology Politehnica Bucharest.	11

*Give information separately for exposed and unexposed groups.

Note: An Explanation and Elaboration article discusses each checklist item and gives methodological background and published examples of transparent reporting. The STROBE checklist is best used in conjunction with this article (freely available on the Web sites of PLoS Medicine at http://www.plosmedicine.org/, Annals of Internal Medicine at http://www.annals.org/, and Epidemiology at http://www.epidem.com/). Information on the STROBE Initiative is available at www.strobe-statement.org.
