# Supplementary material for: Validation of a Questionnaire Assessing the Link Between Affective State and Physical Activity in Adults: A Cross-Sectional Study
Source: J Clin Med. 2025 May 6;14(9):3210. doi: 10.3390/jcm14093210 (PMC12072974; doi:10.3390/jcm14093210)
Supplement: Supplementary file 1 [file jcm-14-03210-s001.zip › File S2.pdf]

Chestionar Stare Afectivă și Activitate Fizică (CSAAF)

Chestionarul a fost conceput pentru a evalua în mod special modul în care diferite stări afective, cum ar fi motivația scăzută, tristețea sau anxietatea, influențează implicarea în activitatea fizică în ultimele două săptămâni.
Fiecare întrebare este evaluată pe o scală Likert de 5 puncte, cu următoarele categorii de scor: 0 – Niciodată, 1 – Rareori, 2 – Uneori, 3 – Frecvent, 4 – Întotdeauna.

Întrebările chestionarului

1. Simțiți că lipsa energiei vă împiedică să faceți activități fizice?
2. Vă pierdeți interesul față de exerciții fizice sau activități pe care le găseați plăcute din cauza stării de spirit?
3. Când vă simțiți trist/ă sau descurajat/ă, vă este greu să începeți sau să mențineți o activitate fizică?
4. Observați că aveți mai puțină motivație pentru a face exerciții fizice atunci când vă simțiți obosit/ă din punct de vedere psihic?
5. Când vă simțiți neliniștit/ă sau stresat/ă, vă este greu să faceți mișcare?
6. Simțiți că activitatea fizică vă obosește mai mult decât înainte, chiar și fără a depune efort intens?
7. Aveți tendința de a evita activitatea fizică atunci când vă simțiți fără speranță sau descurajat/ă?
8. Credeți că dispoziția dvs. scăzută vă face să fiți mai sedentar/ă decât ați dori?
9. Observați că nu vă puteți bucura de activitățile fizice din cauza grijilor sau a autocriticii?
10. Vă simțiți demotivat/ă să faceți mișcare atunci când vă simțiți lipsit/ă de încredere în propria persoană?
11. Simțiți că vă confruntați cu dificultăți de concentrare în timpul exercițiilor fizice din cauza gândurilor negative?
12. Când vă simțiți anxios/anxioasă, vă este greu să vă relaxați în timpul activităților fizice?
13. Vă simțiți vinovat/ă pentru că nu reușiți să mențineți o rutină de exerciții fizice?
14. Participați mai rar la activități fizice în grup din cauza stării dumneavoastră. emoționale?
15. După activitatea fizică, starea de bine dispare repede și reveniți la starea negativă?

Scorare:
0 – 10: impact minim;
11 – 31: impact ușor;
32 – 37: impact moderat;
peste 38: impact sever.

The Affective State and Physical Activity Questionnaire (ASPAQ)

The ASPAQ was specifically designed to assess how various affective states, such as low motivation, sadness, or anxiety, influence engagement in physical activity over the past two weeks.
Each item is rated on a 5-point Likert scale, with the following score categories: 0 – Never, 1 – Rarely, 2 – Sometimes, 3 – Often, 4 – Always.

Questionnaire items

1. Do you feel that a lack of energy prevents you from engaging in physical activities?
2. Do you lose interest in physical exercise or activities you previously enjoyed due to your mood?
3. When you feel sad or discouraged, do you find it difficult to start or maintain physical activity?
4. Do you notice that you have less motivation to exercise when you feel mentally exhausted?
5. When you feel anxious or stressed, do you find it difficult to engage in physical activity?
6. Do you feel that physical activity tires you more than before, even without intense effort?
7. Do you tend to avoid physical activity when you feel hopeless or discouraged?
8. Do you believe that your low mood makes you more sedentary than you would like to be?
9. Do you find it difficult to enjoy physical activities due to worries or self-criticism?
10. Do you feel unmotivated to exercise when you lack self-confidence?
11. Do you struggle to concentrate during physical exercise because of negative thoughts?
12. When you feel anxious, do you find it difficult to relax during physical activities?
13. Do you feel guilty for not being able to maintain an exercise routine?
14. Do you participate less frequently in group physical activities due to your emotional state?
15. After physical activity, does the sense of well-being fade quickly, bringing you back to a negative state?

Scoring:
0 – 10: minimal impact;
11 – 31: mild impact;
32 – 37: moderate impact;
above 38: severe impact.
